# The Effects of Laughter Therapy on General Health of Elderly People Referring to Jahandidegan Community Center in Shiraz, Iran, 2014: A Randomized Controlled Trial

**Published:** 2015-01

**Authors:** Fariba Ghodsbin, Zahra Sharif Ahmadi, Iran Jahanbin, Farkhondeh Sharif

**Affiliations:** 1 Community Based Psychiatric Care Research Center, Department of Community Health Nursing, School of Nursing and Midwifery, Shiraz University of Medical Sciences, Shiraz, Iran;; 2 Department of Geriatric Nursing, School of Nursing and Midwifery, Shiraz University of Medical Sciences, Shiraz, Iran;; 3 Shiraz Geriatric Research Center, Department of Community Health Nursing, Shiraz University of Medical Sciences, Shiraz, Iran;; 4 Community Based Psychiatric Care Research Center, Department of psychiatric Nursing, School of Nursing and Midwifery, Shiraz University of Medical Sciences, Shiraz, Iran

**Keywords:** Elderly, General health, Laughter therapy

## Abstract

**Background:** Aging and its social-biological process naturally impair the functions of different body organs and cause progressive disabilities in managing personal affairs and performing social roles. Laughter therapy is an important strategy which has been recommended by experts for increasing health promotion in older adults. Therefore, we aimed to investigate the effect of laughter therapy program on public health of senior citizens.

**Methods: **In a randomized controlled trial, we enrolled 72 senior citizens aged 60 and over referring to Jahandidegan (Khold-e-Barin) retirement community center in Shiraz, southwest Iran during January to February 2014. The participants were assigned into experimental (N=36) and control (N=36) groups. Data were collected using General Health Questionnaire (GHQ-28) and demographic questionnaire. The participants of experimental group attended a laughter therapy program consisting of two 90-minute sessions per week lasting for 6 weeks.

**Results:** We found a statistically significant correlation between laughter therapy program and factors such as general health (P=0.001), somatic symptoms (P=0.001), insomnia and anxiety (P=0.001). However, there was no statistically significant correlation among laughter therapy, social dysfunction (P=0.28) and depression (P=0.069).

**Conclusion: **We concluded that laughter therapy can improve general health and its subscales in elderly people.

**Trial Registration Number:** IRCT2014061111691N4

## Introduction

The world’s population is rapidly aging and developing countries are more involved with such issue. According to the United Nations’ estimates, elderly population is predicted to increase from almost 10.5% of the total population in 2007 to 28.8% by 2050.^1^ In 2006, 7.3% of whole Iranian population aged 60 and over.^2^

Aging and its social-biological process naturally impair the functions of different body organs and cause progressive disabilities in managing personal affairs and performing social roles. Such impairments are more evident in social and psychological dimensions of the elderly people’s life and may impose limitations on personal and social communications networks. However, social and psychological dimensions of life have been found to influence health significantly.^3^^,^^4^

As the elderly population increases, the health problems, especially mental health problems, of such an age group become more important. Accordingly, the elderly’s health issues can be investigated in two dimensions of physical and psychological. Physical problems such as different types of cancers, cardiovascular diseases and chronic obstructive pulmonary disorders are more prevalent in older adults than their younger counterparts. Depression, anxiety and dementia are also common psychological problems among this age group.^4^ On the other hand, the incidence of psychiatric disorders in senior citizens, who are residents of nursing homes, is approximately 80%. Among the psychological health issues occurring among the aging population, depression has the highest prevalence rate (17%).^5^ Public health in the elderly people includes four dimensions: physical health, physical functioning, anxiety and depression.^6^

For health promotion in older adults, various strategies have been recommended by experts, among which laughter therapy is an important one.^7^ Laughter causes synchronized contraction of facial muscles, increases respiratory rate, blood flow and the release of adrenaline in blood and ultimately leads to joy and happiness. It is the cheapest medicine for preventing many diseases and fighting against them. Laughter also decreases the heart beat rate and blood pressure while it increases oxygen intake in tissues by making the individuals take deep breaths. Hence, laughter can benefit both mental and physical health.^8^^,^^9^

On the condition that psychological stress-induced hormone is secreted continuously and in large amounts, the immune system cannot fully perform its normal functions. Therefore, it puts the internal organs of the body under intense pressure and paves the way for various infections and diseases. Research has proved that laughter plays a critical role in strengthening the immune system and maintaining health and wellness.^10^ Laughter has been indicated to have several positive physiologic effects, one of the important of which is helping the individual cope with stress and reducting anixity; in addition, that may have a role in reducing the incidence of obesity that may positively effect the physical health.^11^

Currently, there are several laughter therapy clubs in various parts of the world where a group of people gather to practice laughter as a form of exercise. In laughter exercise program, initially the participants laugh “artificially” until, gradually, the fake laughter becomes realandleads to releasing anti-stress and joyful hormones. Moreover, in laughing, the diaphragm and abdominal muscles move and stimulate the parasympathetic nervous system; this reduces stress hormones and ultimately promotes relaxation in individuals.^12^^,^^12^

Since special attention should be paid to physical and mental health in older adults, laughter therapy can be applied as an effective strategy leading to health promotion, vitality and happiness in such age group.^13^ Therefore, we aimed to investigate the effect of a 12-week laughter therapy program on public health of the senior citizens referring to Jahandidegan (Khold-e-Barin) retirement community center in Shiraz.

## Methods

This study was approved by the Ethics Committee of Shiraz University of Medical Sciences (Ethics Committee Approval Number: CT-92-6759). In this non-blinded randomized controlled trial (because intervention of laughter the arapycannotbe conducted as blinding), from 1000 records of the elderly registered in Jahandidegan (Khold-e-Barin) 80 were selected through simple random method by their records. The sample size was calculated as 36 in each group based on the data of similar studies and using mean comparison formula (the mean and standard deviatin in the experiment and control groups were 39.5±10 and 32.90±.9.50, respectively with a power of 80% and α: 0.05).^13^

$$n=\frac{{\left({\text{Z}}_{1-\frac{\alpha }{2}}+{\text{Z}}_{1-\beta }\right)}^{2}\left(\delta_{1}^{2}-\delta_{2}^{2}\right)}{{\left(\mu_{1}-\mu_{2}\right)}^{2}}$$

$$\frac{{\left(\text{1.96}+\text{0.85}\right)}^{2}\left(\text{10.4}-\text{9.5}\right)}{{\left(\text{39.5}-\text{32.9}\right)}^{2}}=\text{36}$$

**Figure 1:**
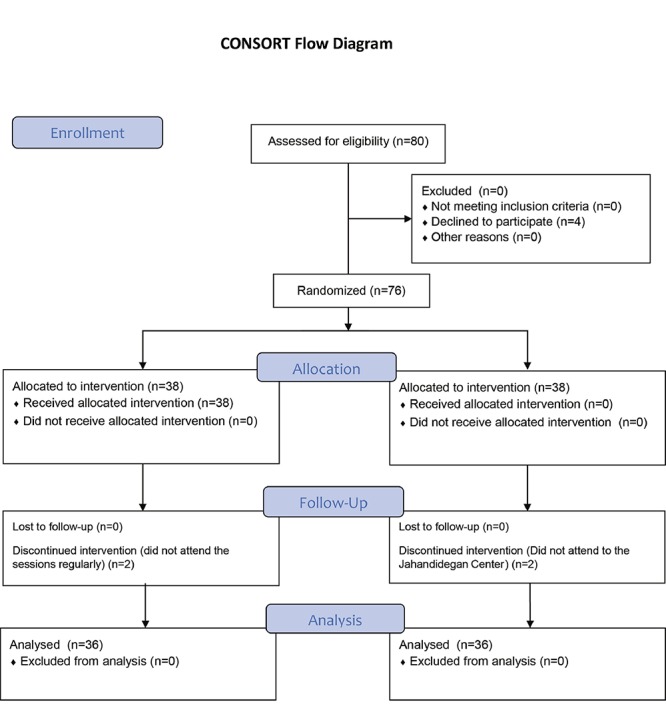
Diagram of the participants in the study

In addition, based on withdrawal cases in the follow up time, sample size was increased to 40 participants in each group. In this study, 80 senior citizens aged 60 and over attended Jahandidegan (Khold-e-Barin) daily community center of aging in shiraz, southwest Iran during January to February 2014. After obtaining written informed consent, balanced block randomization method was used to randomize the participants, In fact, block ranomization was used for selection of samples. All of the four blocks were selected (AABB, ABAB, ABBA, BBAA, BABA, BAAB) and numberd from 1-6 and randomization was done using the tagble of random numbers. Finally 40 elderly people were enrolled in the experimental and control groups. Eight participants were excluded from the study due to different reasons (Four of them were excluded because they did not attend the sessions regularly and 4 of them refused to participate in the study) (Figure 1).

Inclusion criteria were the age of 60 years and over, willingness to participate in the study, completing written informed consent form, not participating in a similar study simultaneously and lack of mental disability. The exclusion criteria were any experience of social-family crisis during the study, unwillingness to participate in the study, the absence of more than two sessions and any types of respiratory diseases which can be transmitted to other participants.

After explaining the aims and method of the research to the participants, data were collected using General Health Questionnaire (GHQ-28) and demographic questionnaire which enquired about age, sex, age, educational, marital and occupational status. GHQ-28 was completed by the participants of the experimental and control groups before and after the intervention.

GHQ-28 is a 28-item self-report questionnaire which contains 4 subscales measuring somatic symptoms, anxiety, insomnia, social dysfunction, and depression. The score for each sub scale ranges from 0 to 21. The total score, which is obtained by summing up the scores of all subscales, ranges from 0 to 84 with lower scores indicating higher general health status. The reliability and validity of the questionnaire were assessed in various studies and were also estimated and confirmed in Iran by Mohammad et al. (2009) and Malakouti et al. (2007) after being translated into Persian. Cronbach’s alpha was calculated as 0.9.^14^^,^^15^

The elderly in the experimental group participated in a laughter therapy program consisting of two 90-minute sessions per week for 6 weeks. The program included performing breathing and physical exercises as well as laughter techniques. However, the participants of the control group received no intervention. The questionnaires were completed again by the participants of both groups immediately after the intervention.

The collected data were analyzed using SPSS software, version 21. Non-parametric tests including Wilcoxon and Mann-Whitney were used. The significance level was set at 0.05. Also, for assessment of normality Kolmogorov–Smirnov test was applied. 

## Results

The mean±SD age of the participants was 68.55±6.24 and 67.27±5.87 in the experimental and control groups, respectively. 66.7% of the participants in the control and 72.2% in the experimental groups were women. Table 1 shows demographic variables of the participants.

**Table 1:** Frequency distribution of demographic variables in the experimental and control groups

**Variables**	**Classification**	**Experimental group** **Frequency (Percentage)**	**Control Group** **Frequency (Percentage)**
Age	60-65	12 (33.3)	18 (50)
	65-70	14 (38.9)	8 (22.2)
	70-75	4 (11.1)	3 (8.4)
	higher than 75	6 (16.7)	7 (19.4)
Ducational Status	Illiterate	6 (16.7)	4 (11.1)
	Primary Education	16 (44.4)	20 (55.6)
	Secondary Education	8 (22.2)	10 (27.8)
	Higher Education	6 (16.7)	2 (5.6)
Marital Status	Single	0 (0)	2 (5.6)
	Married	14 (38.9)	18 (50)
	Divorced	6 (16.7)	4 (11.1)
	Widow	16 (44.4)	12 (33.3)
Occupational Status	Unemployed	12 (33.3)	4 (11.1)
	Self-employed	4 (11.1)	2 (5.6)
	Clerk	0 (0)	2 (5.6)
	Retired	6 (16.7)	12 (33.3)
	Housewife	14 (38.9)	16 (44.4)
Sex	Male	10 (27.8)	12 (33.3)
	Female	26 (72.2)	24 (66.7)

The mean scores of general health and its subscales as well as the correlations between mean scores of subscales before and after the intervention are shown in Table 2. In addition, results of Kolmogorov–Smirnov test (K–S test) showed that data in our study did not have a normal distribution, so non- parametric tests were used for data analysis (P=0.01).

**Table 2:** Comparison of the mean scores of general health subscales before and after the intervention

**Groups**		**Experimental group**			**Control Group**			
**Scales**	**Before the Intervention**		**After the Intervention**	P value*	Before the Intervention^**^	After the Intervention^***^	P value*
	**Mean±SD**		**Mean±SD**		**Mean±SD**	**Mean±SD**	
Somatic symptoms	6.86±4.30		3.47±3.44	0.001	7.05±5.83	4.91±3.40	0.02
Anxiety and insomnia	7.83±4.74		3.84±2.77	0.001	6.55±5.06	4.33±3.22	0.01
Social dysfunction	7.66±4.93		5.25±3.25	0.04	5.72±5.64	3.72±3.44	0.09
Depression	9.27±7.98		4.24±4.20	0.01	5.98±6.25	4.44±4.41	0.01
General health	34.94±21.76		18.33±16.16	0.01	36.80±10.41	20.25±16.52	0.83
								

*Used from Wilcoxon test; ^**^The same time as before the intervention in the experimental group; ^***^The same time as after the intervention in the experimental group

Table 2 shows that the status of general health and all its subscales improved in the experimental group after the intervention. Moreover, the difference between the scores before and after the intervention was statistically significant for all subscales in the experimental group (P=0.05). In the control group, the status of all subscales improved significantly after the intervention (P=0.05), while we found no statistically significant difference between the total mean scores of general health and social dysfunction in such group before and after the intervention.

The differences in the mean scores of the two groups were compared to specify the correlation between laughter therapy and public health and its subscales (Table 3).

**Table 3:** Comparison of the difference in the mean scores of general health and its subscales in the experimental and control groups before and after the intervention

**Public Health Scales**	**Groups**	**Mean Difference (Before-after)**±SD	**Z**	P value*
Somatic Symptoms	Experimental group	-3.39±4.97	-2.05	0.04
	Control Group	-2.13±4.73		
Anxiety and insomnia	Experimental group	-4±5.38	-2.21	0.03
	Control Group	-2.12±4.78		
Social dysfunction	Experimental group	-3.41±6.54	-1.06	0.28
	Control Group	-2±6.09		
Depression	Experimental group	-5.08±8.03	-1.81	0.07
	Control Group	-1.80±6.59		
General Health	Experimental group	-16.61±26.19	-3.57	0.0001
	Control Group	-16.55±10.43		

*Used from Mann-Whitney test

Table 3 shows that the difference between the experimental and control group was statistically significant in terms of the mean scores of general health and other subscales and insignificant in terms of the mean scores of physical dysfunction and depression.

## Discussion

We examined the effect of a laughter therapy program on the public health of the senior citizens referring too neoftheretirement community centers in Shiraz. We compared the mean scores of general health subscales in the experimental and control groups before and after the intervention (Table 2). The results showed that the mean score of somatic symptoms’sub scale significantly decreased in the experimental group after the intervention, indicating the positive effect of intervention on the participants of this group (P=0.001). A significant difference was also detected in the control group between the mean scores in this regards before and after the intervention (P=0.02). Our findings also demonstrated that general health and all its subscales had been improved in the experimental group after the laughter therapy intervention and the improvement was statistically significant. However, we found no significant difference in the control group between the scores of social dysfunction (P=0.09) and general health total scores (P=0.83) before and after the intervention. Laughter decreases stress hormones and increases the immune cells and antibodies; thus, it can be considered as a factor that improves the general health.^16^^-^^19^

Furthermore, we compared the difference in mean scores of general health and its subscales in the experimental and control groups before and after the intervention. The significant difference between the two groups on each subscale is also reported in Table 3. According to our results, the difference in the mean scores of somatic symptoms was found to be statistically significant in both groups (P=0.04), reflecting the effectiveness of the interventions in improvement of this subscale in the participants. Our finding was consistent with those of Behzadi, Ramezani and Saifie Zarie, explaining the positive effect of laughter therapy in improving such subscale by influencing respiration, improving the immune system, relaxing muscles as well as reducing pain, blood pressure and previous health problems.^7^^,^^10^^,^^16^^-^^19^

Besides, we observed that the difference in the mean scores of anxiety and insomnia was statistically significant in both groups (P=0.027), reflecting the effect of the interventions on reducing anxiety and insomnia in our elderly participants. The result was similar to those of Behzadi, Ko et al., Houston et al., Bennett et al., Dixon and and Thorson, signifying the positive effects of laughter therapy on eliminating negative thoughts, changing attitudes and beliefs, creating positive emotional states, discharging dense excitement, and alleviating the symptoms of diseases.^12^^,^^13^^,^^16^^,^^20^^-^^23^

However, the difference in the mean scores of social dysfunction subscale was not statistically significant in both groups (P=0.28), confirming the ineffectiveness of the interventions in improvement of such disorder in the participants. This finding was in contrast with those of Behzadi and Shahidi due to differences in the sample size and duration of the interventions.^13^^,^^16^

Also, the difference in the mean scores of depression subscale was not statistically significant in the experimental and control groups (P=0.04), reconfirming the ineffectiveness of the interventions in reducing depression in the participants. The findings of Behzadi, Ko and Youn and Shahidi were different from ours in this regard. Such difference may have resulted from the short duration of our study and the fact that making changes in depression mood is a timely process. If we could continue the study for a longer time, we would probably observe changes in depression mean scores.^12^^,^^13^^,^^16^

The difference in the mean scores of general health was found to be statistically significant in both groups (P=0.001), signifying the positive effect of laughter therapy intervention on the improvement of general health in the elderly people who participated in our study. Several studies found similar results and also mentioned laughter therapy as the simplest and the most cost-effective method of reducing the mentioned health problems. Our results have also proved the fact that some factors such as participation in group laughter therapy sessions and accompanying peers, sense of solidarity and belonging to the group, creating positive emotional states, changing attitudes and improving relationships with others as well as social relationships could positively affect general health.^16^^,^^22^^,^^24^^,^^25^ Besides, improving mood and cell activity, reducing stress, strengthening the immune system, improving attitudes towards self and life, increasing self-confidence, energy, precision and concentration are among other influential factors that can affect general health.^13^^,^^26^^,^^27^^,^^25^

The advantages of present sudy were its design as RCT that removed the confunder variables and sutible attendance of the elderly with low withdrawal in the time of research. However, the intervention (Laughter therapy) was subjective and was implanted in a short period of time (6 weeks); this was the main limitation of this study. 

## Conclusion

We can conclude that laughter therapy can improve general health and its subscales in the elderly people. Therefore, the results obtained from the present study can help authorities and experts in the field of geriatric care management to adopt precise plans and policies to increase general health by making the senior citizens and their families aware about the advantages of laughter therapy and establishing laughter therapy clubs. 
